# Influence of Ribavirin Serum Levels on Outcome of Antiviral Treatment and Anemia in Hepatitis C Virus Infection

**DOI:** 10.1371/journal.pone.0158512

**Published:** 2016-07-07

**Authors:** Thomas Kuntzen, Sereina Kuhn, Daniela Kuntzen, Burkhardt Seifert, Beat Müllhaupt, Andreas Geier

**Affiliations:** 1 Department of Gastroenterology and Hepatology, University Hospital Zürich, Zürich, Switzerland; 2 Department of Internal Medicine, Kantonsspital Baden, Baden, Switzerland; 3 Epidemiology, Biostatistics and Prevention Institute, University Zürich, Zürich, Switzerland; 4 Department of Internal Medicine II, University Hospital Würzburg, Würzburg, Germany; University of Sydney, AUSTRALIA

## Abstract

**Background:**

Ribavirin blood levels vary considerably between patients with standard weight-based dosing. Their impact on sustained virological response (SVR) with pegylated interferon and ribavirin is controversial, but has mostly been studied before the IL28b gene polymorphism as a possible confounder was discovered.

**Methods:**

The impact of serum ribavirin trough levels at week 4, at the end of treatment and of mean levels across the entire antiviral treatment with pegylated interferon and ribavirin on relapse, SVR rates and anemia was retrospectively studied by univariate and multivariable logistic regression analyses in 214 patients with HCV genotype 1–4 infection, including 88 patients with available IL28b genotyping.

**Results:**

Mean ribavirin levels varied between 0.68–5.65 mg/l and significantly differed between patients with or without SVR. By multivariable regression including age, sex, HCV viral load, HCV genotype, liver fibrosis stage, prior treatments, immunosuppression and IL28b genotype, ribavirin levels consistently displayed significant influence on SVR and relapse without indication for a specific importance of higher concentrations early or late in the treatment course. Although hemoglobin decline was on average more pronounced in patients with higher ribavirin levels, hemoglobin remained relatively stable in a significant proportion of these, indicating that ribavirin levels alone are insufficient to predict anemia.

**Conclusion:**

While data are scarce to draw conclusions applicable for modern DAA therapies, these results support ribavirin treatment based on serum levels instead of purely weight-based dosing in combination with pegylated interferon.

## Introduction

Pegylated interferon and ribavirin have been the standard treatment for Hepatitis C virus (HCV) infection for more than a decade and are still in use in many developing countries. Although new directly acting antiviral drugs (DAAs) inhibiting the viral protease, polymerase and the non-structural protein 5a have enormously improved efficacy and tolerability approaching 100% cure rates in patients with favorable characteristics predicting treatment response [1], patients with liver cirrhosis or HCV genotype 3 infection remain difficult to treat populations where ribavirin is still a key drug to improve SVR rates [2–6]. Thus, despite anemia as a frequent side effect, ribavirin continues to be part of modern antiviral treatment regimens.

In combination with pegylated interferon, guidelines recommended weight-based ribavirin dosing between 1000–1200 mg/d for HCV genotypes 1 and 4, and a fixed dose of 800 mg/d for treatment of HCV genotypes 2 and 3 [5, 7]. However, ribavirin plasma levels vary considerably between patients even with weight-based dosing [8]. Since ribavirin has a narrow therapeutic range with anemia as a frequent side effect, monitoring of plasma levels might be useful to optimize treatment outcomes and avoid unnecessary toxicity. Multiple studies described a benefit of higher ribavirin doses on sustained virological response (SVR) rates, but are challenged by almost equal numbers of studies reporting contradictory results [9]. Interpretation is limited (critically reviewed in [9]) by differences in patient populations and study end points, often small sample sizes, and the fact that only some of the few, mostly retrospective studies that measured plasma or serum ribavirin levels consistently obtained these as trough levels as necessary at least for the investigation of the first 4–8 weeks of treatment until steady state levels are established [10]. Last, most publications came out before the IL28b gene polymorphism was discovered as one of the strongest factors influencing response rates with pegylated interferon and ribavirin therapy, thus representing a possible confounder for all past analyses regarding the effect of ribavirin. Currently, only five studies considering the IL28b gene polymorphism—three of them on HIV/HCV coinfected patients—have been published. While the two publications on HCV mono-infected patients favored higher ribavirin levels, conflicting results were observed in the HIV/HCV co-infected cohorts. However, each study focused on special patient subgroups, ribavirin dosing strategies or sample time points [11–15]. Thus, it is still unclear whether ribavirin concentrations might be relevant and could warrant therapeutic drug monitoring in anti-HCV treatment.

We therefore conducted a retrospective study on the influence of ribavirin serum concentrations on SVR and relapse rates considering IL28b genotypes, as well as on anemia in patients treated for HCV genotype 1–4 infection with pegylated interferon and ribavirin at the University Hospital Zürich between 2005 and 2013.

## Patients and Methods

### Study Population

Adult Caucasian patients who had received anti HCV treatment between July 2005 and June 2013 were retrospectively screened for the study. Inclusion criteria were infection with HCV genotypes 1–4, treatment with pegylated interferon and ribavirin with known outcome, and availability of serum ribavirin trough level measurements. Exclusion criteria were non-standard treatment durations or interferon dose reductions preventing assessment of the treatment response. The only HIV positive patient in the cohort achieved SVR, so that HIV infection appeared no relevant confounder in this case and the patient was kept in the study. The study was conducted according to the Declaration of Helsinki and current guidelines on Good Clinical Practice and was approved by the Cantonal Ethics Committee Zürich, that waived the need for individual patient consent acknowledging the non-interventional and retrospective character of the study. Patient information was anonymized and de-identified prior to the statistical analysis.

### Treatment Regimens and Assessment of Response

Patients were treated with pegylated interferon alpha 2a or 2b and ribavirin at weight-based dosages and for treatment durations according to standard guidelines [5, 7]. Anemia was managed with ribavirin dose reduction, erythropoietin or transfusions as necessary, and in some cases the ribavirin dose was increased in patients with low plasma ribavirin through levels at the discretion of the treating physician. Treatment was considered sufficient if patients had completed at least 90% of the recommended treatment duration without interferon dose reduction. In patients with pre-treatment viral load < 800.000 IU/ml and undetectable HCV RNA at treatment week 4 (rapid virological response, RVR) treatment could be shortened from 48 to 24 weeks for HCV genotypes 1 and 4, or from 24 to 16 weeks for HCV genotypes 2 and 3. Patients who did not achieve a two log reduction in their viral load at treatment week 12 (early virological response, EVR) were considered non-responders and treatment was discontinued. An undetectable HCV RNA 24 weeks after the end of treatment was considered a sustained virological response (SVR).

#### Non-standard treatment durations

An extension of the treatment duration by 24 weeks was allowed in patients with negative predictors including cirrhosis, failed prior treatment with pegylated interferon and ribavirin, or immunosuppression after transplantation [5, 7], but was considered as an influencing factor in the multivariate analysis to avoid confounding effects. Significantly (>10%) shorter than recommended treatment durations were observed because of early discontinuations in case of intolerable side effects, receipt of a transplant organ, newly diagnosed cancer, infections, death or loss to follow up. Patients who nevertheless achieved SVR were counted as responders. All other patients with non-standard treatment durations that did not allow classification of their treatment response were excluded from the analysis.

### Measurements and Laboratory Tests

Patients were followed using a standardized treatment schedule with regular visits and standardized lab sheets including ribavirin trough level measurement in weeks 4, 12, 24 and 48, and at additional time points as felt necessary by the treating physician. Serum ribavirin trough levels were measured using high-performance liquid chromatography tandem mass spectrometry using a validated and accredited method by the hospital’s central clinical chemistry lab, and mean values from all available time points were calculated using the linear trapezoid rule. If the ribavirin dosage was changed during the treatment course, patients were only included in the study if at least one subsequent ribavirin measurement reflecting the change was available. Liver fibrosis was assessed according to the Metavir score [16] either from pre-treatment liver biopsies, or from fibroscan measurements with conversion to Metavir scores with cutoff levels according to Castera et al. [17]. HCV RNA levels were determined using Cobas Ampliprep Taqman HCV quantitative tests (Roche Diagnostics, Switzerland). IL28b genotyping for SNP rs12979860 was performed using the LightMix Kit IL28b (Tib Molbiol, Berlin, Germany) on a Roche LightCycler (Roche Diagnostics, Switzerland).

### Statistical Analysis

Continuous variables were compared by Mann-Whitney-U test and categorical variables by chi-square test or Fisher’s exact test as appropriate using SPSS software version 23 (IBM Corp., Armonk, NY). Variables with significant results in the univariate analysis and ribavirin levels were entered into a logistic regression analysis to evaluate independent factors influencing SVR in the entire study population, or in subgroups of patients based on HCV genotypes and availability of IL28b genotyping. P-values < 5% were considered significant.

## Results

### Study Population

To analyze the impact of ribavirin serum levels and IL28b genotypes together with other known influencing factors on anti-HCV treatment response rates, a total of 276 patients were retrospectively screened. Of these, 214 patients had sufficient ribavirin measurements and information on treatment outcome, as well as HCV genotype, liver fibrosis stage, baseline HCV RNA levels, treatment duration, prior treatments, sex, age and immunosuppression as previously described factors influencing treatment outcome [5, 7] available and could be included applying the inclusion and exclusion criteria. IL28b genotyping was available for 88 patients (54 for genotypes 1 and 4, and 34 for genotypes 2 and 3). SVR was achieved by 43/77 (56%) patients with genotype 1, 22/26 (85%) patients with genotype 2, 59/82 (72%) patients with genotype 3 and 15/29 (52%) patients with genotype 4. Patient characteristics and SVR rates are displayed in Tables 1–3.

**Table 1:** Patient characteristics and SVR rates.

	All HCV Genotypes			
	N (All)	SVR	non-SVR	p
**N**	214	139	75	
**Age, mean (range)**	214	44.9 (20–72)	50.5 (25–69)	**0.001**
**Sex**				**0.025**
male, n (% SVR)	144	86 (60%)	58 (40%)	
female, n (% SVR)	70	53 (76%)	17 (24%)	
**Log**_**10**_ **HCV-RNA, mean (range)**	214	5.7 (2.7–7.4)	6.2 (4.7–7.3)	**0.002**
**Metavir Fibrosis Score, n**				**0.002**
F0-1, n (% SVR)	80	60 (75%)	20 (25%)	
F2, n (% SVR)	62	39 (63%)	23 (37%)	
F3, n (% SVR)	27	13 (48%)	14 (52%)	
F4, n (% SVR)	29	11 (38%)	18 (62%)	
missing	16	16 (100%)	0 (0%)	
**Treatment status, n (%)**				**< 0.001**
Treatment naive	170	125 (73.5%)	45 (26.5%)	
Treatment experienced	44	14 (32%)	30 (68%)	
Standard Interferon ± Ribavirin	26	6 (23%)	20 (77%)	
Pegylated Interferon + Ribavirin	18	8 (44%)	10 (56%)	
**Immunosuppression, n (%)**				0.58
Yes	25	15 (60%)	10 (40%)	
No	189	124 (66%)	65 (34%)	
**Extended treatment duration, n (%)**				0.84
Yes	27	18 (67%)	9 (33%)	
No	187	121 (65%)	66 (35%)	
**IL28b rs12979860**				0.85
CC, n (%)	28	16 (57%)	12 (43%)	
CT/TT, n (%)	60	33 (55%)	27 (45%)	
missing, n	126			
**Ribavirin serum level (mg/l), mean (range)**				
Week 4	171	2.4 (0.6–6.7)	2.0 (0.2–5.3)	**0.015**
End of treatment	113	2.7 (0.0–6.2)	2.3 (0.5–4.6)	**0.024**
Mean, full treatment duration	214	2.7 (0.7–5.7)	2.4 (0.9–5.3)	**0.045**

Patient characteristics and cofactors analyzed regarding treatment outcome with rates of sustained virological response (SVR) for all patients. P-values of univariate analyses are indicated. (Mann-Whitney-U Test, Chi-Squared Test or Fisher's exact Test were used as appropriate.)

**Table 2:** Patient characteristics and SVR rates for HCV Genotypes 1 and 4.

	HCV Genotype 1 and 4			
	N (All)	SVR	no SVR	p
**N**	106	58	48	
**Age, mean (range)**	106	45.4 (20–71)	50.1 (25–69)	**0.008**
**Sex**				0.35
male, n (% SVR)	77	40 (52%)	37 (48%)	
female, n (% SVR)	29	18 (62%)	11 (38%)	
**Log**_**10**_ **HCV-RNA, mean (range)**	106	5.7 (3.0–7.4)	6.2 (4.7–7.2)	**0.005**
**Metavir Fibrosis Score, n**				**0.001**
F0-1, n (% SVR)	43	30 (70%)	13 (30%)	
F2, n (% SVR)	35	20 (57%)	15 (43%)	
F3, n (% SVR)	17	7 (41%)	10 (59%)	
F4, n (% SVR)	10	0 (0%)	10 (100%)	
missing	1	1 (100%)	0 (0%)	
**Treatment status, n (%)**				**0.006**
Treatment naive	72	46 (64%)	26 (36%)	
Treatment experienced	34	12 (35%)	22 (65%)	
Standard Interferon ± Ribavirin	21	4 (19%)	17 (81%)	
Pegylated Interferon + Ribavirin	13	8 (61.5%)	5 (38.5%)	
**Immunosuppression, n (%)**				0.66
Yes	18	9 (50%)	9 (50%)	
No	88	49 (56%)	39 (44%)	
**Extended treatment duration, n (%)**				1.00
Yes	8	4 (50%)	4 (50%)	
No	98	54 (55%)	44 (45%)	
**IL28b rs12979860**				0.29
CC, n (%)	17	10 (59%)	7 (41%)	
CT/TT, n (%)	37	16 (43%)	21 (57%)	
missing, n	52			
**Ribavirin serum level (mg/l), mean (range)**				
Week 4	77	2.7 (0.7–6.7)	2.3 (0.2–5.3)	**0.049**
End of treatment	57	3.0 (0.0–6.2)	2.5 (0.5–4.6)	**0.047**
Mean, full treatment duration	106	3.0 (0.8–5.7)	2.8 (0.9–5.3)	0.13

Patient characteristics and cofactors analyzed regarding treatment outcome with rates of sustained virological response (SVR) separately for patients infected with HCV genotypes 1 and 4.

**Table 3:** Patient characteristics and SVR rates for HCV Genotypes 2 and 3.

	HCV Genotype 2 and 3			
	N (All)	SVR	no SVR	p
**N**	108	81	27	
**Age, mean (range)**	108	44.5 (21–72)	49.7 (38–68)	0.05
**Sex**				0.11
male, n (% SVR)	66	46 (70%)	20 (30%)	
female, n (% SVR)	42	35 (83%)	7 (17%)	
**Log**_**10**_ **HCV-RNA, mean (range)**	108	5.8 (2.7–7.2)	6.1 (4.9–7.3)	0.10
**Metavir Fibrosis Score, n**				0.26
F0-1, n (% SVR)	37	30 (81%)	7 (19%)	
F2, n (% SVR)	27	19 (70%)	8 (30%)	
F3, n (% SVR)	10	6 (60%)	4 (40%)	
F4, n (% SVR)	19	11 (58%)	8 (42%)	
missing	15	15 (100%)	0 (0%)	
**Treatment status, n (%)**				**< 0.001**
Treatment naive	98	79 (81%)	19 (19%)	
Treatment experienced	10	2 (20%)	8 (80%)	
Standard Interferon ± Ribavirin	5	2 (40%)	3 (60%)	
Pegylated Interferon + Ribavirin	5	0 (0%)	5 (100%)	
**Immunosuppression, n (%)**				0.68
Yes	7	6 (86%)	1 (14%)	
No	101	75 (74%)	26 (26%)	
**Extended treatment duration, n (%)**				1.00
Yes	19	14 (74%)	5 (26%)	
No	89	67 (75%)	22 (25%)	
**IL28b rs12979860**				0.43
CC, n (%)	11	6 (55%)	5 (45%)	
CT/TT, n (%)	23	17 (74%)	6 (26%)	
missing, n	74			
**Ribavirin serum level (mg/l), mean (range)**				
Week 4	94	2.1 (0.6–4.5)	1.6 (0.7–3.0)	**0.005**
End of treatment	56	2.4 (0.6–4.3)	1.8 (0.5–2.7)	**0.007**
Mean, full treatment duration	108	2.4 (0.7–4.3)	1.9 (1.1–2.9)	**0.001**

Patient characteristics and cofactors analyzed regarding treatment outcome with rates of sustained virological response (SVR) for patients infected with HCV genotypes 2 and 3.

### Variability of Serum Ribavirin Levels

Serum ribavirin concentrations were measured a median of 3 times (mean 3.33, range 1–7) during treatment, with intrapatient mean values calculated over the entire treatment duration ranging between 0.68 and 5.65 mg/l. The median intrapatient variability was 16% (IQR 42%, range 0–49%) in patients with constant ribavirin dosage throughout the treatment course and at least two available measurements, and slightly higher (19%, IQR 55%, range 3–86%) in patients with dose adjustments. Comparing mean ribavirin serum levels between patients with different HCV genotypes and treatment responses, these varied between 1.9 mg/l and 3.0 mg/l (Tables 1–3). As demonstrated in previous reports [8, 11–13] these values illustrate the considerable inter-individual variability in ribavirin plasma levels with standard ribavirin dosing.

### Factors Influencing Sustained Virological Response

Multiple studies have investigated factors that influence the outcome of antiviral treatment with pegylated interferon and ribavirin [5, 7]. By univariate analysis including the full dataset, consistent with previous reports Metavir fibrosis scores, HCV RNA levels, age, sex, HCV genotype and prior unsuccessful treatment showed significant differences between patients with or without SVR, whereas—possibly because of the relatively low numbers of patients with these characteristics in our cohort—immunosuppression, extended treatment duration and IL28b CC versus non-CC genotype did not reach significance. Regarding the impact of ribavirin, mean levels over the entire treatment duration and ribavirin levels at week 4 and at the end of treatment were significantly different between patients achieving SVR and those who could not be cured (Table 1). A separate analysis grouping patients with genotypes 1/4 or 2/3 yielded similar results, with the only exception that mean ribavirin levels over the entire treatment duration did not reach significance in the genotype 1/4 subgroup (Tables 2 and 3).

Variables with significant results by univariate analysis were entered into a logistic regression model. Here, liver fibrosis, HCV viral load, ribavirin levels at all three analyzed time points, HCV genotype and prior unsuccessful treatment were still associated with SVR, whereas age and sex failed the level of significance. When the analysis was restricted to patients with available IL28b genotyping to include this variable in the model, mean ribavirin levels over the full treatment period or at week 4, prior unsuccessful treatment and baseline HCV viral load remained significant factors influencing SVR rates (Table 4).

**Table 4:** Multivariate logistic regression analysis of factors influencing sustained virological response.

Variable	All patients (N = 198)				Patients with available IL28b genotype (N = 81)			
	β	OR	95% CI	p-value	β	OR	95% CI	p-value
Mean ribavirin serum level	1.0	2.7	1.7–4.3	**< 0.001**	1.2	3.3	1.4–7.8	**0.006**
Treatment-naive	1.9	6.6	2.9–15.0	**< 0.001**	2.3	10.4	2.5–42.7	**0.001**
Fibrosis (Metavir score)	-0.4	0.7	0.5–1.0	**0.035**	-0.5	0.6	0.3–1.3	0.18
HCV genotype 2/3 (vs. 1/4)	1.3	3.5	1.6–7.8	**0.002**	0.7	1.9	0.5–8.2	0.38
Log_10_ HCV-RNA	-0.4	0.6	0.5–0.9	**0.006**	-0.4	0.7	0.4–1.1	0.13
Age	-0.2	1.0	0.95–1.02	0.33	-0.05	1.0	0.9–1.0	0.20
Female gender	0.2	1.2	0.6–2.6	0.64	0.7	2.0	0.5–7.7	0.30
IL28b CC (vs. CT/TT)		-	-	-	0.1	1.1	0.3–3.8	0.90

Results for the multivariate regression analysis with mean ribavirin serum levels over the full treatment period. Similar results were obtained using ribavirin levels from treatment week 4. Due to missing liver fibrosis scores or IL28b genotyping, the analysis is restricted to 198 and 81 patients with complete data for all factors included in the analysis. The regression coefficient β as well as odds ratios with confidence intervals are shown.

Taken together, both by univariate and by multivariate analysis including important described covariables, ribavirin serum levels turned out to be one of the strongest factors significantly influencing the outcome of anti-HCV treatment with pegylated interferon and ribavirin in our study.

### Factors Influencing Relapse

Some previous studies have focused on the role of ribavirin concentrations at specific time points such as week four or the end of treatment, with a recent publication indicating that higher end of treatment ribavirin levels might prevent virological relapse [9, 12]. To evaluate whether high drug concentrations might indeed be of special importance at certain time points during treatment, we carried out a focused analysis to assess relapse rates in relation to ribavirin levels.

Among 181 patients with end of treatment response, 43 experienced virological relapse– 23/81 (28%) with genotype 1/4 and 20/100 (20%) with genotype 2/3. By univariate analysis, liver fibrosis scores (p = 0.015), HCV viral load (p<0.001), age (p = 0.015) and ribavirin levels at the end of treatment (p = 0.036) differed significantly between relapsers and sustained responders, and relapses occurred significantly more often in treatment experienced patients (p<0.001), whereas HCV genotype, extended treatment duration, immunosuppression, IL28b genotype and ribavirin levels at week 4 or mean levels over the entire treatment duration showed no detectable effect.

Since ROC analyses have identified a plasma threshold around 2 mg/l of ribavirin as an optimal cutoff level to distinguish responders from non-responders in several studies [12, 18, 19], we also analyzed relapse rates in patients with ribavirin levels above or below 2 mg/l over the entire treatment period, among which we compared patients where ribavirin levels changed above or below 2 mg/l at the end of treatment. Of 135 patients with mean ribavirin levels > 2 mg/l, 7 patients had ribavirin levels < 2 mg/l at the end of treatment, all of which achieved SVR, whereas relapse occurred in 25/128 (19.5%) patients with ribavirin levels stably above 2 mg/l during and by the end of treatment (p = 0.2). In the remaining 46 patients with mean ribavirin levels below 2 mg/l, relapse rates were again not significantly different between patients with persistently lower ribavirin levels also by the end of treatment (7/17; 41%) compared to patients in whom ribavirin levels rose above 2 mg/l at the end of treatment (11/29; 38%; p = 0.8).

In the multivariate regression model including all factors already analyzed for SVR rates above, only previous unsuccessful treatment and ribavirin levels at all analyzed time points (week 4, end of treatment, and mean ribavirin levels over the entire treatment duration) showed significant influence on relapse rates. The findings remained significant when IL28b was included in the model, whereby the analysis was restricted to 56, 32 and 67 patients with available IL28b genotyping for the week 4, end of treatment and entire treatment duration time points, respectively (data not shown).

Thus, our results support higher drug concentrations in general as a significant factor influencing both SVR and relapse, rather than indicating a special importance of high ribavirin levels at specific time points during the treatment course.

### Impact of Ribavirin Serum Levels on Anemia

Previous studies on dual treatment with pegylated interferon and ribavirin found target concentrations of 10–15 μM (2.44–3.66 mg/l) ribavirin optimal for balancing high SVR rates with anemia as the most important ribavirin side effect [20], which is a particular concern still limiting its use also in the era of DAA regimens.

To evaluate whether therapeutic drug monitoring might aid in the prevention of anemia, we analyzed hemoglobin decline as a function of mean ribavirin serum concentrations in groups with a hemoglobin decline of more or less than 3 g/dl. Patients on dialysis were excluded because of the need for anemia management influencing hemoglobin levels independent of ribavirin treatment in this group. As expected, significantly higher rates of hemoglobin decline > 3 mg/dl were observed with higher ribavirin concentrations (p = 0.015), illustrating that optimal drug levels for maximum antiviral efficacy must be balanced with drug toxicity. Naturally, in none of the patients with hemoglobin decline less than 3 g/dl ribavirin dose reduction was necessary. Notably, however, less than 3 g/dl hemoglobin decline occurred in 23–27% of patients with high ribavirin serum levels of > 3 mg/l and up to 5.3 mg/l (Table 5).

**Table 5:** Proportion of patients with hemoglobin decline ≤ 3 mg/l or > 3 mg/l in groups of increasing mean serum ribavirin concentrations.

Mean ribavirin concentration	Hemoglobin decline	
	≤ 3 g/dl	> 3 g/dl
	n (%)	n (%)
**0–2.00 mg/l**	28 (51%)	27 (49%)
**2.01–3.00 mg/l**	27 (31%)	61 (69%)
**3.01–4.00 mg/l**	11 (23%)	37 (77%)
**> 4.00 mg/l**	4 (27%)	11 (73%)

Thus, ribavirin serum levels appeared unsuitable for the prediction of its hemolytic effect to guide ribavirin dosing.

## Discussion

In the present multivariate analysis of factors influencing the outcome of anti-HCV treatment with pegylated interferon and ribavirin, especially also after considering IL28b genotypes we found a significant influence of ribavirin serum levels both on SVR and relapse rates, remarkably with consistently stronger impact compared to other patient characteristics such as older age or HCV RNA levels. This effect was not limited to early or late time points during treatment, arguing against a special importance of ribavirin only for specific treatment phases. Although overall anemia was more pronounced in patients with higher ribavirin concentrations, hemoglobin levels remained relatively stable in 23–27% of these, indicating that preemptive strategies avoiding high ribavirin levels might lead to unnecessary under-treatment in a relevant proportion of patients.

To date, only five studies that also included IL28b genotyping have investigated the role of ribavirin levels for the outcome of anti HCV treatment. In HCV mono-infected patients, higher ribavirin levels were associated with lower relapse rates [12] and higher SVR rates [11], whereat the effect of higher ribavirin concentrations remarkably overcame racial disparities in African Americans with unfavorable IL28b genotypes, who achieved SVR rates similar to Caucasian patients [11]. On the other hand, in HIV/HCV co-infected patients only Torres-Cornejo described a positive influence of higher ribavirin levels on SVR rates in patients with non-favorable IL28b genotypes [13], whereas two other studies failed to detect a significant influence on treatment outcome [14, 15], raising the question whether the effect of ribavirin might be attenuated in the HIV co-infected population that generally achieves lower SVR rates with pegylated interferon and ribavirin compared to HCV mono-infected patients.

While our results are in line with the two studies on HCV mono-infected patients, as a limitation shared with most publications we cannot exclude possible biases due to the retrospective nature of our study. In addition we acknowledge that the influence of the IL28b gene polymorphism could not be demonstrated in our cohort. A shift in SVR rates up to 40% [21] between different IL28b genotypes was described for HCV genotypes 1 and 4, but has shown controversial results in HCV genotypes 2 and 3 in medium-sized datasets [22], perhaps because of a weaker IL28b impact in these genotypes requiring large cohorts for clarification [23]. Therefore, with only 106 genotype 1 or 4 and 108 genotype 2 or 3 infected patients, respectively, our study was likely underpowered for the detection of the influence of IL28b. However, based on our results it should also be noted that none of the studies focusing on IL28b took ribavirin concentrations into account, which may explain conflicting results regarding the role of IL28b in patients with HCV genotype 2 or 3 infection [22, 23].

At present there are only three studies available on the influence of ribavirin concentrations on SVR rates in the context of DAAs: One analyzed data from trials with interferon, ribavirin and the protease inhibitors telaprevir or boceprevir, the second studied HCV genotype 1 infected patients that received low dose (600 mg) or weight-based (1000–1200 mg) ribavirin in combination with sofosbuvir for 24 weeks, and the third combined patients with HCV genotype 3 infection treated with sofosbuvir and ribavirin in the Fission, Positron and Fusion trials. In all three studies higher ribavirin levels were associated with higher SVR rates [24–26]. While these single DAA regimens have been superseeded by more potent treatment options, ribavirin is currently still recommended for difficult to treat patients with HCV genotype 1 infection even when powerful DAA combinations such as sofosbuvir and ledipasvir or ombitasvir, ritonavir, paritaprevir and dasabuvir are used [1]. The high SVR rates of 97–100% achieved with these regimens may leave little space for optimization by ribavirin dose adjustment. However, lower SVR rates are achieved in the treatment of HCV genotype 2 infected patients with sofosbuvir and ribavirin for 12–16 weeks (60–91%), as well as in the treatment of HCV genotype 3 with pegylated interferon, ribavirin and sofosbuvir for 12 weeks (83–91%), or with ribavirin and sofosbuvir without interferon for 24 weeks (30–92%), especially if negative predictors such as cirrhosis or prior unsuccessful treatment are present [1]. Since a clear advantage of higher ribavirin levels has recently been shown for sofosbuvir and ribavirin treatment for HCV genotype 3 [26], it would be interesting to know whether individualized ribavirin dosing might be useful to optimize treatment response rates in some of these settings.

Hemolytic anemia as the most important ribavirin side effect generally limits dose escalations. Due to the retrospective nature of our study we could not analyze anemia and its management in detail because different thresholds for ribavirin dose reduction, transfusion or use of erythropoietin were applied by different physicians, preventing statistical comparisons. However, even by only focusing on patients with a relatively mild hemoglobin decline < 3 g/dl that did not require anemia management we could demonstrate that a significant proportion of these individuals tolerated high ribavirin levels of 3–5.3 mg/l. On the other hand, anemia has been described to develop with ribavirin concentrations as low as 5–8 μM (1.22–1.95 mg/l) [20]. Therefore, currently ribavirin levels seem to be of little value to predict anemia, for which hopefully studies e.g. on polymorphisms in the inosine triphosphatase (ITPA) [27, 28] or the SLC28A2 [29] genes will enable a more precise view in the future. While ribavirin target concentrations of 2.44–3.66 mg/l for dual treatment with pegylated interferon, 2.2–3.6 mg/l for pegylated interferon, ribavirin and either telaprevir or boceprevir [25], or 4.4–6 pmol/10^6^ red blood cells for Sofosbuvir plus ribavirin [24] have been published as optimal concentration ranges to balance the chances for SVR with the rate of anemia < 10 g/dl, we therefore rather suggest that ribavirin levels of at least 2.5–4 mg/l but without a strict upper limit should be targeted to increase SVR rates, while anemia should be managed by ribavirin dose reduction, erythropoietin or transfusion on an individual basis when it occurs. The feasibility of higher dosing combined with monitoring of drug levels to reach target concentrations above 2 mg/l has been demonstrated in several trials. Different strategies with either calculation of the optimal ribavirin dose based on weight and renal function [30–33], or the use of a pre-treatment ribavirin test-dose were applied [34], whereby the proportion of patients that would only achieve inadequately low ribavirin exposure using standard weight-based dosing may be reduced considerably. Notably, in a study comparing an intensified regimen with 2000 mg ribavirin per day for the first four weeks of treatment against normal weight based ribavirin dosing, the decline in hemoglobin could well be compensated by erythropoietin [14]. Given that DAA treatment durations are as short as 8–12 weeks, it appears well possible that anemia may be manageable for this limited time even with higher ribavirin dosing.

Taken together, we here demonstrate a beneficial effect of higher ribavirin concentrations in HCV infected patients treated with pegylated interferon and ribavirin. For countries where peg-interferon and ribavirin dual therapy is still first-line treatment our results may be of direct value to improve outcomes.

## Supporting Information

S1 Table Ribavirin_Data. Raw data analyzed for this study.
(XLSX)
